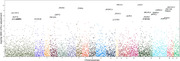# Increasing the power of rare variant association analyses with common variant eQTLs in Alzheimer’s disease

**DOI:** 10.1002/alz.093228

**Published:** 2025-01-03

**Authors:** Yuchen Yang, William S. Bush

**Affiliations:** ^1^ Case Western Reserve University, Cleveland, OH USA; ^2^ Department of Population and Quantitative Health Sciences, Institute for Computational Biology, Case Western Reserve University, Cleveland, OH USA

## Abstract

**Background:**

Many independent studies have found rare variants associated with AD. Current gene‐based tests for rare‐variants generally consider the impact of low‐frequency coding variants as an independent effect from the common regulatory variants that surround them. In this work, we propose to increase the statistical power of kernel‐based rare‐variant association tests by accounting for the surrounding cis‐regulatory variants’ effects on gene expression. We intend to identify novel AD associations of genes with rare variants on existing haplotype backgrounds.

**Method:**

We incorporate common variant cis‐eQTLs around the gene or SNP‐set of interest as a fixed effect by estimating their impact on gene expression using GTEx association models. The Sequence Kernel Association Test (SKAT) is then used to test for the residual random effect of coding rare‐variants (MAF<1%). SKAT is known to retain statistical power when a small proportion of test variants are causal, and when these effects are in mixed directions. We perform comprehensive simulation studies that demonstrate the biological conditions under which our method can substantially increase power, including a range of effect sizes for gene expression, providing insight into the relationship between eQTLs and rare variants. Power analyses were performed with both exact p‐values computed via the Davies method and derived empirically through permutation tests.

We then applied our approach to data from the Alzheimer’s Disease Sequencing Project to evaluate gene‐level associations across 6461 genes with 210,000 missense rare variants. We also extend this approach to other set‐based rare variant association tests such as SKAT‐O and variant‐set mixed model association tests.

**Result:**

Simulations show that our method can substantially increase power, while maintaining the type I error in the absence of association. Our proposed method improves the power of SKAT where a low proportion (<10%) of tested variants have small mixed effect sizes (OR<1.0), closely resembling real world variant effects. We confirm previously reported significant associations such as TREM2, and find several novel gene candidates that required this adjustment to be study‐wide significant.

**Conclusion:**

Our approach is useful for discovering rare variation in genes that contribute to AD risk and heritability. This method can be flexibly incorporated into many existing analyses pipelines.